# Different type of pay later facility for green product with selling price dependent demand using grey wolf optimizer

**DOI:** 10.1016/j.heliyon.2024.e32398

**Published:** 2024-06-04

**Authors:** Adel Fahad Alrasheedi

**Affiliations:** Department of Statistics and Operations Research, College of Science, King Saud University, P.O. Box 2455, Riyadh, 11451, Saudi Arabia

**Keywords:** Green product, Inventory model, Partial and full trade credit facility, Green level dependent purchase cost, GWO algorithm

## Abstract

The use of trade credit finance is becoming more widely acknowledged as a crucial approach to improving inventory system profitability. We review an inventory model with depending on permitted payment delays for which, if the retailer place an orders higher than or equal to a predefined quantity $S_{1}$, then the supplier will provide a fully pay in later facility of ξ periods (i.e., there will be no charge of interest until ξ). On the other hand the retailer need to pay a partial amount of payment to the supplier if the order quantity is less than $S_{1}$, and the remaining amount may be deferred for up to ξ periods. Main objective of this study is to investigate the inventory model with different situations under delayed payment facility. In addition, determining the product's demand also involves taking into account the item's greenness and selling price. We have also considered the fact that the cost of buying is influenced by the product's degree of greenness. We employ the meta heuristic algorithm Grey Wolf Optimizar (GWO) to assist us in solving the problem, and we compare the outcomes with the aid of a few other algorithms (Whale optimisation algorithm (WOA) and Artificial electric field algorithm (AEFA)). In the end, we resolve several numerical cases to support the model. The concavity of the desired function is graphically displayed using MATLAB software.

## 1 Introduction

Green products are goods or services that have been designed and manufactured with an emphasis on reducing their environmental impact. They are also referred to as environmentally friendly or sustainable products. Generally speaking, green products are made with as little waste as possible and as little pollution as possible throughout their lifecycle. These goods are made to satisfy consumer demands without having a negative impact on the environment, supporting conservation and sustainability initiatives. Energy-efficient appliances, environmentally friendly cleaning supplies, electric vehicles, and organic food items are all examples of green products. As people try to live more sustainably and become more conscious of the effects their actions have on the environment, there has been a rise in the demand for green products in recent years.

A trade credit facility is a type of financial agreement that enables companies to give suppliers credit terms for goods and services. A trade credit facility is essentially a kind of short-term loan that suppliers give to their clients, enabling the latter to settle payments for products and services at a later time, usually within a few weeks or months. Trade credit facilities are widespread in sectors like wholesale, retail, and manufacturing. The supplier and the customer negotiate the terms of the credit facility, including the interest rate, payment schedule, and credit limit. The credit limit denotes the highest credit amount that the supplier is prepared to offer the client.

Harris [1] proposed the economic order quantity (EOQ) in 1913. Since, academics have created a vast number of adaptations to the inventory system. When the seller proposes a reasonable payment delay, Goyal [2] developed an EOQ inventory model for the buyer. A torrent of studies have been coming out of Goyal's [2] model in recent years, making it one of the foundational works in the subject of trade credit. Subsequently, Shah [3] takes into account a stochastic inventory model when payment delays are acceptable. Jamal et al. [4] then expand Goyal [2]'s approach to incorporate shortages. Hwang and Shinn [5], who simultaneously incorporate the pricing strategy into the model to jointly calculate the appropriate price and quantity for a retailer under the supposition of a legal payment delay. Moreover, it should be noted that Teng [6] offers an alternate discussion to Goyal [2] and demonstrates that it is economically sensible for a seasoned buyer to place fewer orders and benefit from permitted delays more rapidly. Chang et al. [7] create a supplier credit EOQ model for deteriorating products tied to purchasing amount one year later. Huang [8] builds on Goyal's [2] by expanding it to produce an EOQ model, in which the supplier provides the retailer with up-stream trade payment term M and the retailer then provides the customer with the downstream trade credit period N. (with NM). Huang [8] model, nonetheless, has some shortcomings. Ouyang et al. [9,10] proposed two deteriorating inventory model under different type of credit policy. Goyal et al. [11] investigated the best ordering practises in cases if the provider provides a rising interest-paying plan. Teng and Goyal [12] offer a generalised formulation to address Huang [8]'s model's flaws in this application. The initial proponent, Huang [13], suggests an EOQ model with conditionally acceptable payment delays, if the merchant purchases much more or equal to a set amount W, the supplier offers a fully permitted delay of M periods. The merchant must, however, pay the supplier a part of the total purchased amount instantly if the order is less than W, while enjoying an M-period wait on the rest amount. The model is accurate and useful. His mathematical formulations and graphical representations of the income earned and the interest charged, however, can sometimes be misleading. In order to address some of the model's flaws, we extend Huang's [13] model in this study. Chang et al. [14] performed an evaluation of trade credit-covered inventory lot-size models. Ho et al. [15] presented a supplier-buyer integrated two-part trade credit inventory model with the most competitive pricing, shipping, and payment terms. Huang and Hsu [16] investigated an economic order quantity model for supply chain purposes under merchant partial pay later facility. Liao [17] introduced a two-level trade credit order quantity model with no instantaneous receiving and exponential rate decaying items. Soni and Shah [18] choosing the best prices, shipping options, and payment terms for a two-part trade credit supplier-buyer inventory model. Furthermore, Teng [19] develops an inventory system for a retailer that provides different pay later facility to its clients with excellent and bad credit. Chang et al. [20] then present the best manufacturer replenishment strategies in a supply chain with upstream and downstream trade credits. Kreng and Tan [21,22] introduced two different inventory model under different type of credit facility. Teng et al. [23] found that when the supplier gives a progressive allowable payment delay, the merchant has the best ordering policy. The proper manner inventory model proposed by Lin et al. [24] assumes that both the supplier and the retailer employ trade credit policies and that the retailer receives some damaged goods. In contrast, the inventory models stated above implicitly assume that each item was delivered to the shop in pristine condition. If the supplier grants a legitimate payment delay, Cheng et al. [25] take into account various financial situations. Teng and Lou [26] investigated a supply chain involving upstream and downstream trade credits, the seller's ideal credit duration and replenishment time should be considered. Jaggi et al. [27] recently developed a novel inventory model for items of subpar quality with acceptable shortages and allowable payment delays. Pal et al. [28] suggested a supply chain model under different credit facility. Ouyang et al. [29] suggested a capacity restriction inventory system with credit facility dependent on order size of the product. Yang et al. [30] investigated decaying inventory system with preservation facility under credit policy approach. Wu et al. [31] investigated a model for degrading items with a freshness related product under trade agreement credits to customers who pose a significant credit risk. Bhunia and Shaikh [32] proposed a credit policy approach in an alternative way in the area of inventory and solved the said problem with the help of particle swarm optimisation. Shaikh [33,34] suggested two different inventory model with different types of credit facility. Puliafito et al. [35] investigated the energy sector of Argentina's high-resolution atmospheric emission inventory. Evaluation against the EDGAR Global Emission Database. Tiwari et al. [36] investigated a joint impact on pricing and deterioration with credit facility under backlogged situations. Shaikh et al. [37] presented a trade credit inventory model with three parameters, where the deterioration rate is governed by a Weibull distribution. According to Cárdenas-Barrón et al. [38], holding costs under credit facilities and nonlinear stock-dependent demand are both recommended. Shaikh et al. [39] studied ramp-type demand-related inventory model with preservation, and a credit facility was provided. In the latter facility, Das et al. [40] investigated the preservation inventory model in conjunction with multiple pay. Das et al. [41] proposed the credit facility approach in a production system. Rahman et al. [42] combined a facility for advance payment with an inventory requirement. Bassey and Zelibe [43] proposed a Model for two-echelon inventory location that incorporates lateral transshipment and response time requirements. Taleizadeh et al. [44] introduced a carbon emission related inventory model with credit facility. Das et al. [45] introduced a inventory model for green products along with credit facility. Dissa and Ushakumari [46] proposed an inventory system consisting of two commodity queueing, two demand classes, a pool of customers, and a random common lifetime. Jeganathan et al. [47] discussed replacing unsuccessful items in a queueing-inventory system with two commodities that retrials with multi-component demand and vacation interruption. Sun et al. [48] investigated an important update on the list of landslides that were caused by the August 8, 2017, Mw6.5 Jiuzhaigou earthquake in China, along with assessments of their spatial distribution.

A class of computing methods named as "soft computing" is designed in order to solve problems that are difficult to solve with conventional methods. Algorithms for soft computing are frequently utilised in situations when there is a notable degree of uncertainty or ambiguity in the data, or when the data is complex and difficult to express. Soft computing algorithms find widespread use in various domains, including robotics, image processing, data mining, control systems, and decision support systems. They are especially useful when more adaptable and flexible solutions are needed, or when traditional procedures don't work.

It is possible to differentiate three types of meta-heuristics: algorithms based on physics, evolutionary theory, and swarm intelligence. On the other hand, the algorithms developed based on evolutionary strategy are grounded in the natural selection of principles and are utilised for search and optimisation problems, swarm intelligence algorithms are modelled after the collective behaviour of social insects. In this area, genetic algorithm (GA) is the most widely used algorithm. This method models the ideas of Darwnian evolution, and it was proposed by Holland [49]. Goldberg [50] conducted a thorough investigation into the engineering uses of GA. In EAs, optimisation is frequently accomplished by progressively enhancing a random initial solution. Every new population is created by combining and altering the individuals from the preceding generation. The likelihood of the best individuals contributing to the creation of the next generation means that it will probably be superior to the one before it (s). This can ensure that over several generations, the initial random population is improved. Differential evolution (DE) (Storm and Price [51], evolutionary programming (EP) (Yao et al. [52], evolution strategy (ES) (Hansen et al. [53], Fogel [54]), biogeography-based optimizer (BBO) (Simon [55]) and genetic programming (GP) (Koza [56], Rechenberg [57]) are a few EAs.

The second main topic of meta-heuristics consists of methods based on physics. These optimisation strategies typically mimic physical rules. The Gravitational Search Algorithm (GSA) algorithm, developed by Rashidi et al. [58] in 2009, the Big-Bang Big-Crunch (BBBC) algorithm, developed by Erol and Eksin [59] in 2006, Artificial Chemical Reaction Optimisation Algorithm (ACROA) proposed by Alatas [60], Galaxy-based Search Algorithm (GbSA) developed by Shah-Hosseini [61], the Gravitational Local Search (GLSA) algorithm, developed by Webster and Bernhard [62] in the year 2003, Black Hole (BH) introduced by Hatamlou [63], Ray Optimisation (RO) introduced by Kaveh and Khayatazad [64] in the year 2012, Curved Space Optimisation (CSO) suggested by Moghaddam et al. [65], the Central Force Optimisation (CFO) algorithm are introduced by Formato [66] in the year 2007, Small-World Optimisation Algorithm (SWOA) studied by Du et al. [67], and the Charged System Search (CSS) algorithm is developed by Kaveh and Talatahari [68] in the year 2010. In contrast to EAs, these algorithms employ a random assortment of search agents that interact and move about the search space in line with physical laws. For instance, gravitational force, ray casting, electromagnetic force, inertia force, weights, and other forces are used to perform this movement.

**Fig. 1:**
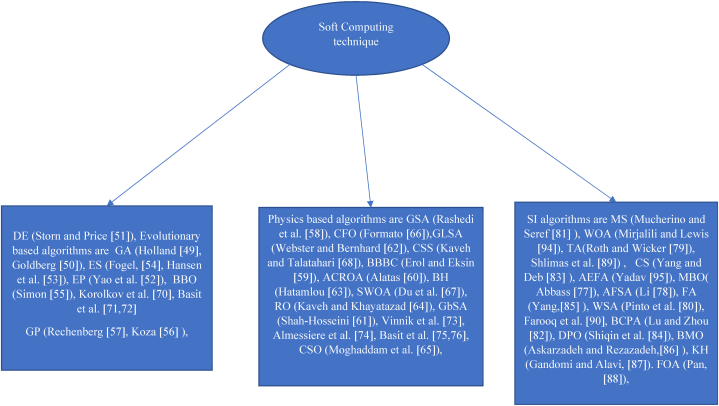
Survey of the literature on various algorithms.

The third category of meta-heuristics are swarm intelligence (SI) techniques. The behavioural patterns of swarms, herds, flocks, or schools of living things in the natural world are basically mimicked by these algorithms. The searchers move around by modelling the social and collective intelligence of animals, even though their method is essentially physics-based. Particle swarm optimisation (PSO) is the most popular algorithm in SI approach. Kennedy and Eberhart [69] suggested the PSO algorithm, which was modelled after the flocking of birds. Several particles that follow both the position of the best particle and their own current best places are used in the PSO method. Stated differently, while a particle is being moved, consideration is given to both its optimal solution and the optimal solution discovered by the swarm. In adition, few algorithms based on swarm intelligent, evolutionary algorithms and physics based algorithms are worth mentioneing viz. Korolkov et al. [70], Basit et al. [[71], [72]], Vinnik et al. [73], Almessiere et al. [74], Basit et al. [[75], [76]], Abbass [77], Li [78], Roth and Wicker [79], Pinto et al. [80], Mucherino and Seref [81], Lu and Zhou [82], Yang and Deb [83], Shiqin et al. [84], Yang [85], Askarzadeh and Rezazadeh [86], Gandomi and Alavi [87], Pan [88], Shlimas et al. [89], Farooq et al. [90], Mirjalili and Lewis [94] and Yadav [95]. Fig. 1 shows a graphic representation of a few SI algorithms, physics-based algorithms, and evolutionary algorithms.

This list illustrates how many search- and hunt-related behaviours have inspired the several SI tactics that have been presented thus far.

The importance of trade credit financing as a strategy for increasing inventory management profitability is becoming more widely acknowledged. The supplier permits a retailer to order more than or equal to a predetermined quantity, and the supplier authorises a completely authorised delay of periods. This is an example of an economic order quantity model with conditionally authorised payment delays. $S_{1}$. (i.e., there is no interest charge until ξ). If the order quantity is less than $S_{1}$, the retailer must make a partial payment to the supplier; the balance may be postponed for up to ξ weeks. We enhance the EOQ outlined previously under conditionally acceptable payment delays in this study to overcome several model defects. We use the Grey wolf Optimizar (GWO) meta-heuristic algorithm to tackle the issue. Finally, we resolve a number of numerical cases to support the concept. Using MATLAB software, the goal function's concavity is presented graphically.

## 2 Notation and assumptions

The following notation and presumptions are introduced in order to develop an EOQ model.

### Notation

The following parameters and variables are used to build the problem.

**Table:** 

Notation	Definition
$D\left(s_{p},\mu \right)$	Demand that is depending on nonlinear green levels and selling price (units)
K	Ordering cost
$c_{1},c_{2}$	Purchasing cost related parameters
$c_{p}$	Unit purchasing cost (units)
λ,σ,τ	Parameters related with demand
$c_{h}$	Inventory carrying cost cost/unit
$\varphi_{c}$	Interest charge rate
$\varphi_{e}$	Interest earn rate
T	Cycle length
ξ	Credit period offered by the supplier to retailers
ν	Fraction amount allowed for delayed payment
$T_{S_{1}}$	Time at which threshold amount reaches to zero level.
$S_{1}$	Threshold amount for determining full delayed payment or partly allowed for delay in payment.
TP	The system's overall profit ($)
S	Retailers order quantity (unit)
*TCC*	Total expense of capital
ATP	System's average profit ($/year)
Decision variables:	
T	The buyer's replenishment cycle length expressed in time units (year)
$s_{p}$	The item's selling price ($/unit)

### 2.1 Assumptions

i. Product demand is frequently impacted by a number of factors, such as the product's selling price and degree of greenness. Businesses looking to maximise their pricing strategies and improve the environmental attractiveness of their products must comprehend this relationship. The amount a buyer must pay to acquire a thing is known as the selling price. Price elasticity of demand is a crucial aspect that affects consumer demand, whereby higher prices typically result in reduced demand. A product's environmental features, such as its use of recycled materials, energy efficiency, low carbon footprint, or eco-friendly packaging, are referred to as its "green level." Greener products tend to appeal more to people that care about the environment. Here it is assumed that the relationship between a product's demand and price, green level of the product and mathematically it can be represented as:

$D\left(s_{p},\mu \right)=\lambda -\sigma s_{p}+\tau \mu^{\upsilon }$ where λ,σ,τ,υ>0.

ii. The delayed payment facility, sometimes referred to as trade credit or deferred payment conditions, is a type of financial agreement that permits the buyer to postpone paying for the goods or services they have ordered instead of doing so right away. This facility can have a big impact on the operations of both the seller and the buyer and is a standard practice in many sectors. The supplier will offer a fully acceptable delay in payments if the retailer's placed an order $S\geq S_{1}$. The retailer is therefore permitted to pay the full purchase price $c_{p}S$ at the end of the permitted delay ξ. On the other hand, if the retailers ordered amount $S<S_{1}$ then the retailer is required to pay a portion of the price of $\left(1-\nu \right)c_{p}S$ right away and must pay the remaining amount of $\nu c_{p}S$ at the conclusion of the allowable delay ξ.
iii. An infinite time horizon is taken into account with negligible little lead time.
iv. As soon as the allowable delay has passed, retailer is charged the prime rate by the supplier. $\varphi_{c}$. Conversely, the retailer is free to spend the sales revenues in the stock market or in the production of new products and earn $\varphi_{e}$ return on investment during the allowable delay period.
v. For both consumers and businesses, the cost of purchasing a product based on its level of environmental friendliness is a crucial factor. A product's cost can be greatly impacted by its "green level," which is defined as its environmental features, such as energy efficiency, low carbon footprint, recyclable materials, or environmentally friendly packaging. A product's green rating denotes how environmentally friendly it is. Higher green scores typically indicate that the product was made with more consideration for sustainability and with environmentally friendly materials and procedures. The amount spent to obtain the product is known as the purchasing cost. Numerous factors, such as the price of raw materials, manufacturing procedures, adherence to regulations, and consumer demand, might affect this. The product's purchase price is also considered to be its green level. In mathematical terms, it can be expressed as follows:

$c_{p}=c_{1}+c_{2}\mu^{\gamma }$ where $c_{1},c_{2},\gamma >0$.

vi. Deterioration and shortages are not allowed.

## 3 Mathematical formulation

This model is developed based on the above-mentioned assumptions in the retailers’ point of view. According to the trade credit facility, following situations may take into account.

(i) Situation 1: $T_{S_{1}}\leq \xi$ and (ii) Situation 2: $T_{S_{1}}>\xi$

**1:**$T_{S_{1}}\leq \xi$.

In this situation, related cost and revenue are calculated in the following ways.

**Fig. 2:**
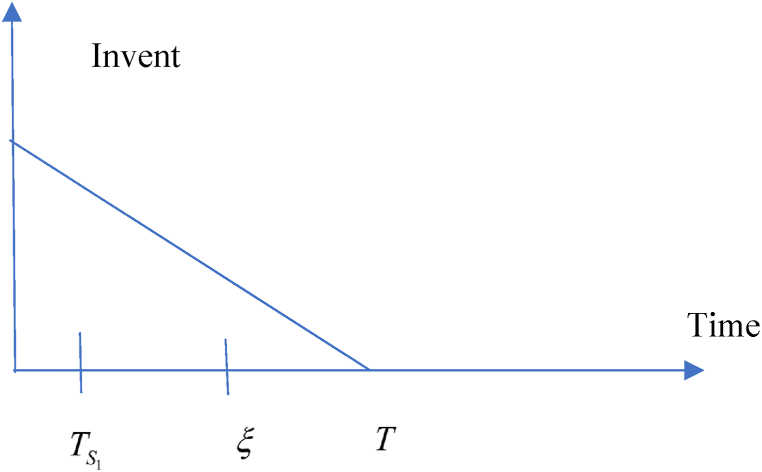
Graphical presentation of sub-situation 1.1.

**Fig. 3:**
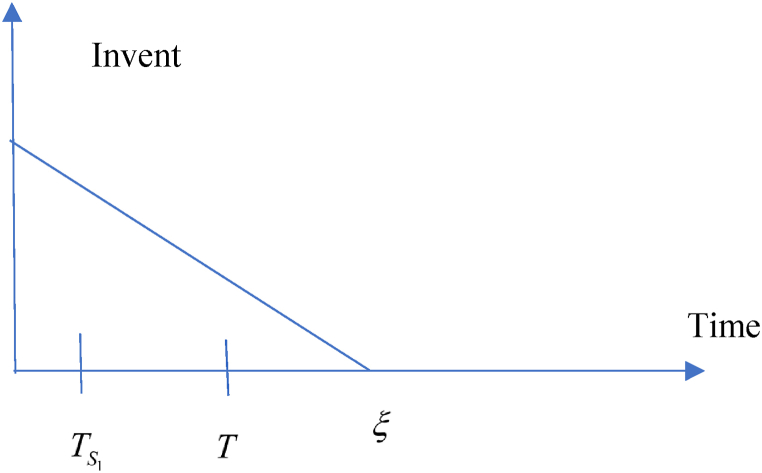
Graphical presentation of sub-situation 1.2.

**Fig. 4:**
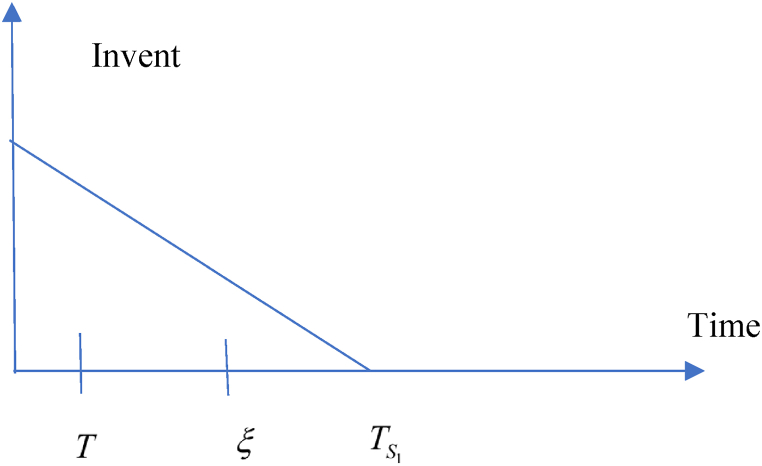
Graphical presentation of sub-situation 1.3.

**Fig. 5:**
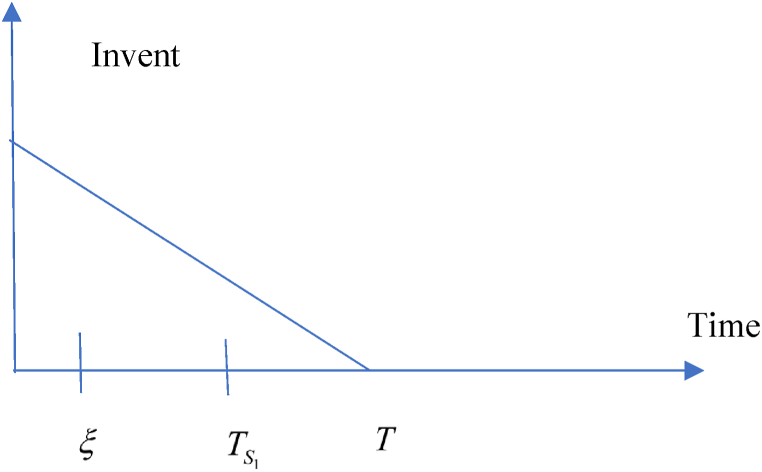
Graphical presentation of sub-situation 2.1.

**Fig. 6:**
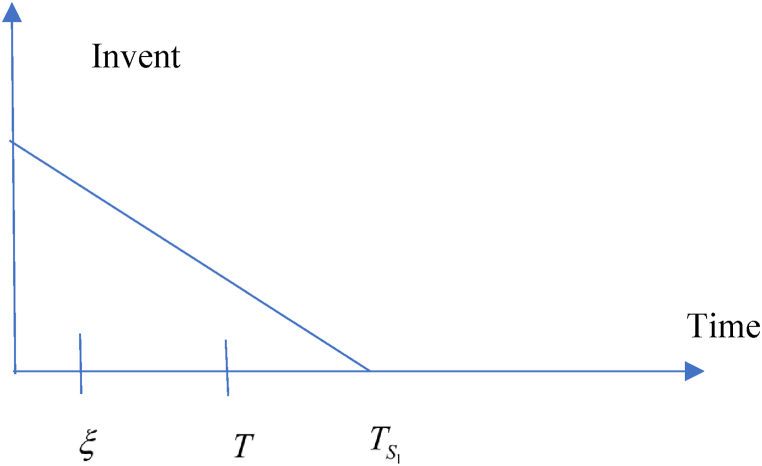
Graphical presentation of sub-situation 2.2.

**Fig. 7:**
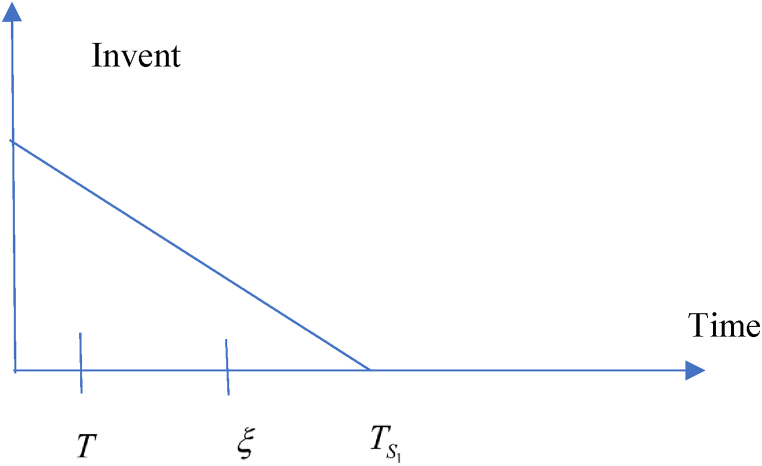
Graphical presentation of sub-situation 2.3.

(i) During the cycle, sales revenue is $\left(SR\right)=s_{p}\left(\lambda -\sigma s_{p}+\tau \mu^{\upsilon }\right)$.
(ii) Inventory carrying cost during the cycle is $\left(HC\right)=\frac{c_{h}\left(\lambda -\sigma s_{p}+\tau \mu^{\upsilon }\right)T}{2}$.
(iii) Cost of ordering (OC) = $\frac{K}{T}$
(iv) Due to the possibility that the replenishment cycle time T could take one of three distinct positions between $T_{S_{1}}$ and ξ, there are three different sub-situations for the annual capital cost. The sub situations may define in the following ways (i) sub-situation 1.1: $T_{S_{1}}\leq \xi <T$ (ii) sub-situation 1.2: $T_{S_{1}}\leq T\leq \xi$ (iii) sub-situation 1.3: $T\leq T_{S_{1}}\leq \xi$ and each situations are given in details. All the calculations are followed from Chen et al. (2014) and different situations are shown from Fig. 2, Fig. 3, Fig. 4, Fig. 5, Fig. 6, Fig. 7.

**sub-situation** 1.1: $T_{S_{1}}\leq \xi <T$.

In this situation, fully credit facility will receive by the retailers from their suppliers. Interest earn throughout the cycle by the retailers is given by $\frac{s_{p}\varphi_{e}\left(\lambda -\sigma s_{p}+\tau \mu^{\upsilon }\right)\xi^{2}}{2T}$. On the other hand interest paid to the entire cycle on capital is given by $\frac{c_{p}\varphi_{c}\left(\lambda -\sigma s_{p}+\tau \mu^{\upsilon }\right){\left(T-\xi \right)}^{2}}{2T}$. Therefore, total capital cost is represented by

$$TCC=\frac{c_{p}\varphi_{c}\left(\lambda -\sigma s_{p}+\tau \mu^{\upsilon }\right){\left(T-\xi \right)}^{2}-s_{p}\varphi_{e}\left(\lambda -\sigma s_{p}+\tau \mu^{\upsilon }\right)\xi^{2}}{2T}\text{.}$$

Therefore, the objective function may be expressed as in equation (1)

(1) $$ATP_{1.1}\left(s_{p},T\right)=\left[\begin{array}{c} s_{p}\left(\lambda -\sigma s_{p}+\tau \mu^{\upsilon }\right)-\frac{c_{h}\left(\lambda -\sigma s_{p}+\tau \mu^{\upsilon }\right)T}{2}\\ -\frac{K}{T}-\frac{c_{p}\varphi_{c}\left(\lambda -\sigma s_{p}+\tau \mu^{\upsilon }\right){\left(T-\xi \right)}^{2}-s_{p}\varphi_{e}\left(\lambda -\sigma s_{p}+\tau \mu^{\upsilon }\right)\xi^{2}}{2T} \end{array}\right]$$

The following is a mathematical representation of the associated optimisation problem in equation (2):

(2) $$\begin{array}{c} Maxmize\;ATP_{1.1}\left(s_{p},T\right)\\ subject\;to\;s_{p}>0,T>0 \end{array}$$

**sub-situation** 1.2: $T_{S_{1}}\leq T<\xi$

this sub situation, fully credit facility will receive by the retailers from their suppliers along with no charge of interest paid. So, interest earn throughout the cycle by the retailers is given by $\frac{s_{p}\varphi_{e}T\left(\lambda -\sigma s_{p}+\tau \mu^{\upsilon }\right)\left(2\xi -T\right)}{2T}$ and interest paid amount will be zero. Therefore, total capital cost is given by $TCC=\frac{0-s_{p}\varphi_{e}T\left(\lambda -\sigma s_{p}+\tau \mu^{\upsilon }\right)\left(2\xi -T\right)}{2T}$.

Therefore, the objective function may be expressed as in equation (3)

(3) $$ATP_{1.2}\left(s_{p},T\right)=\left[\begin{array}{c} s_{p}\left(\lambda -\sigma s_{p}+\tau \mu^{\upsilon }\right)-\frac{c_{h}\left(\lambda -\sigma s_{p}+\tau \mu^{\upsilon }\right)T}{2}\\ -\frac{K}{T}+\frac{s_{p}\varphi_{e}T\left(\lambda -\sigma s_{p}+\tau \mu^{\upsilon }\right)\left(2\xi -T\right)}{2T} \end{array}\right]$$

The following is a mathematical representation of the associated optimisation problem in equation (4):

(4) $$\begin{array}{c} Maxmize\;ATP_{1.2}\left(s_{p},T\right)\\ subject\;to\;s_{p}>0,T>0 \end{array}$$

**sub-situation** 1.3: $T<T_{S_{1}}\leq \xi$

In this sub situation, retailer will receive partial credit facility from their suppliers. In this context payment can be divided into two part, (i) instant payment part (ii) partial credit payment facility of the rest amount of the total purchase cost. For instant payment amount retailers need to pay an interest of the amount $\frac{\left(1-\nu \right)c_{p}\varphi_{c}\left(\lambda -\sigma s_{p}+\tau \mu^{\upsilon }\right)T^{2}}{2T}$ and for delayed payment, there is no interest charged. On the other hand, retailers will earn interest from the partial credit facility with the rate of interest $\varphi_{e}$ and total accumulated amount will be $\frac{\nu s_{p}\varphi_{e}\left(\lambda -\sigma s_{p}+\tau \mu^{\upsilon }\right)T\left(2\xi -T\right)}{2T}$ throughout the entire cycle. Therefore, total capital cost is given by $TCC=\frac{\left(1-\nu \right)c_{p}\varphi_{c}\left(\lambda -\sigma s_{p}+\tau \mu^{\upsilon }\right)T^{2}-\nu s_{p}\varphi_{e}\left(\lambda -\sigma s_{p}+\tau \mu^{\upsilon }\right)T\left(2\xi -T\right)}{2T}$.

Therefore, the objective function may be expressed as in equation (5)

(5) $$ATP_{1.3}\left(s_{p},T\right)=\left[\begin{array}{c} s_{p}\left(\lambda -\sigma s_{p}+\tau \mu^{\upsilon }\right)-\frac{c_{h}\left(\lambda -\sigma s_{p}+\tau \mu^{\upsilon }\right)T}{2}\\ -\frac{K}{T}-\frac{\left(1-\nu \right)c_{p}\varphi_{c}\left(\lambda -\sigma s_{p}+\tau \mu^{\upsilon }\right)T^{2}-\nu s_{p}\varphi_{e}\left(\lambda -\sigma s_{p}+\tau \mu^{\upsilon }\right)T\left(2\xi -T\right)}{2T} \end{array}\right]$$

The following is a mathematical representation of the associated optimisation problem in equation (6):

(6) $$\begin{array}{c} Maxmize\;ATP_{1.3}\left(s_{p},T\right)\\ subject\;to\;s_{p}>0,T>0 \end{array}$$

Situation 2: $T_{S_{1}}>\xi$.

According to $T_{S_{1}},\xi$ and T, three sub-situations may arise in the following ways (i) **sub-situation** 2.1: $\xi <T_{S_{1}}\leq T$ (ii) **sub-situation** 2.2: $\xi \leq T<T_{S_{1}}$ (iii) **sub-situation** 2.3: $T\leq \xi <T_{S_{1}}$. All the situations are described in details and calculation is followed as per Chen et al. (2014).

**sub-situation** 2.1: $\xi <T_{S_{1}}\leq T$.

In this situation, fully credit facility will receive by the retailers from their suppliers. Interest earn throughout the cycle by the retailers is given by $\frac{s_{p}\varphi_{e}\left(\lambda -\sigma s_{p}+\tau \mu^{\upsilon }\right)\xi^{2}}{2T}$. On the other hand interest paid to the entire cycle on capital is given by $\frac{c_{p}\varphi_{c}\left(\lambda -\sigma s_{p}+\tau \mu^{\upsilon }\right){\left(T-\xi \right)}^{2}}{2T}$. Therefore, total capital cost is given by $TCC=\frac{c_{p}\varphi_{c}\left(\lambda -\sigma s_{p}+\tau \mu^{\upsilon }\right){\left(T-\xi \right)}^{2}-s_{p}\varphi_{e}\left(\lambda -\sigma s_{p}+\tau \mu^{\upsilon }\right)\xi^{2}}{2T}$.

Therefore, the objective function may be expressed as in equation (7)

(7) $$ATP_{2.1}\left(s_{p},T\right)=\left[\begin{array}{c} s_{p}\left(\lambda -\sigma s_{p}+\tau \mu^{\upsilon }\right)-\frac{c_{h}\left(\lambda -\sigma s_{p}+\tau \mu^{\upsilon }\right)T}{2}\\ -\frac{K}{T}-\frac{c_{p}\varphi_{c}\left(\lambda -\sigma s_{p}+\tau \mu^{\upsilon }\right){\left(T-\xi \right)}^{2}-s_{p}\varphi_{e}\left(\lambda -\sigma s_{p}+\tau \mu^{\upsilon }\right)\xi^{2}}{2T} \end{array}\right]$$

The following is a mathematical representation of the associated optimisation problem in equation (8):

(8) $$\begin{array}{c} Maxmize\;ATP_{2.1}\left(s_{p},T\right)\\ subject\;to\;s_{p}>0,T>0 \end{array}$$

**sub-situation** 2.2: $\xi \leq T<T_{S_{1}}$

The retailer in this scenario will get a partial credit line from the suppliers. In this instance, the payment can be split into two parts viz. (i) instant payment part (ii) partial credit payment facility of the rest amount of the total purchase cost. For instant payment amount retailers need to pay an interest of the amount $\frac{\left(1-\nu \right)c_{p}\varphi_{c}\left(\lambda -\sigma s_{p}+\tau \mu^{\upsilon }\right)T^{2}}{2T}$ and interest charged for delayed payment to the entire cycle is given by $\frac{\nu c_{p}\varphi_{c}\left(\lambda -\sigma s_{p}+\tau \mu^{\upsilon }\right){\left(T-\xi \right)}^{2}}{2T}$. On the other hand, retailers will earn interest from the partial credit facility with the rate of interest $\varphi_{e}$ and total accumulated amount will be $\frac{\nu s_{p}\varphi_{e}\left(\lambda -\sigma s_{p}+\tau \mu^{\upsilon }\right)\xi^{2}}{2T}$ throughout the entire cycle. Therefore, total capital cost is given by $TCC=\frac{\left(1-\nu \right)c_{p}\varphi_{c}\left(\lambda -\sigma s_{p}+\tau \mu^{\upsilon }\right)T^{2}+\nu c_{p}\varphi_{c}\left(\lambda -\sigma s_{p}+\tau \mu^{\upsilon }\right){\left(T-\xi \right)}^{2}-\nu s_{p}\varphi_{e}\left(\lambda -\sigma s_{p}+\tau \mu^{\upsilon }\right)\xi^{2}}{2T}$.

Therefore, the objective function may be expressed as in equation (9)

(9) $$ATP_{2.2}\left(s_{p},T\right)=\left[\begin{array}{c} s_{p}\left(\lambda -\sigma s_{p}+\tau \mu^{\upsilon }\right)-\frac{c_{h}\left(\lambda -\sigma s_{p}+\tau \mu^{\upsilon }\right)T}{2}-\frac{K}{T}\\ -\frac{\left(1-\nu \right)c_{p}\varphi_{c}\left(\lambda -\sigma s_{p}+\tau \mu^{\upsilon }\right)T^{2}+\nu c_{p}\varphi_{c}\left(\lambda -\sigma s_{p}+\tau \mu^{\upsilon }\right){\left(T-\xi \right)}^{2}-\nu s_{p}\varphi_{e}\left(\lambda -\sigma s_{p}+\tau \mu^{\upsilon }\right)\xi^{2}}{2T} \end{array}\right]$$

The following is a mathematical representation of the associated optimisation problem in equation (10):

(10) $$\begin{array}{c} Maxmize\;ATP_{2.2}\left(s_{p},T\right)\\ subject\;to\;s_{p}>0,T>0 \end{array}$$

**sub-situation** 2.3: $T\leq \xi <T_{S_{1}}$

In this sub situation, retailer will receive partial credit facility from their suppliers. In this context payment can be divided into two part, (i) instant payment part (ii) partial credit payment facility of the rest amount of the total purchase cost. For instant payment amount retailers need to pay an interest of the amount $\frac{\left(1-\nu \right)c_{p}\varphi_{c}\left(\lambda -\sigma s_{p}+\tau \mu^{\upsilon }\right)T^{2}}{2T}$ and for delayed payment, there is no interest charged. On the other hand, retailers will earn interest from the partial credit facility with the rate of interest $\varphi_{e}$ and total accumulated amount will be $\frac{\nu s_{p}\varphi_{e}\left(\lambda -\sigma s_{p}+\tau \mu^{\upsilon }\right)T\left(2\xi -T\right)}{2T}$ throughout the entire cycle. Therefore, total capital cost is given by $TCC=\frac{\left(1-\nu \right)c_{p}\varphi_{c}\left(\lambda -\sigma s_{p}+\tau \mu^{\upsilon }\right)T^{2}-\nu s_{p}\varphi_{e}\left(\lambda -\sigma s_{p}+\tau \mu^{\upsilon }\right)T\left(2\xi -T\right)}{2T}$.

Therefore, the objective function may be expressed as in equation (11)

(11) $$ATP_{2.3}\left(s_{p},T\right)=\left[\begin{array}{c} s_{p}\left(\lambda -\sigma s_{p}+\tau \mu^{\upsilon }\right)-\frac{c_{h}\left(\lambda -\sigma s_{p}+\tau \mu^{\upsilon }\right)T}{2}\\ -\frac{K}{T}-\frac{\left(1-\nu \right)c_{p}\varphi_{c}\left(\lambda -\sigma s_{p}+\tau \mu^{\upsilon }\right)T^{2}-\nu s_{p}\varphi_{e}\left(\lambda -\sigma s_{p}+\tau \mu^{\upsilon }\right)T\left(2\xi -T\right)}{2T} \end{array}\right]$$

The following is a mathematical representation of the associated optimisation problem equation (13):

(12) $$\begin{array}{c} Maxmize\;ATP_{2.3}\left(s_{p},T\right)\\ subject\;to\;s_{p}>0,T>0 \end{array}$$

The Grey Wolf Optimizer (GWO) is an optimisation algorithm that draws inspiration from nature and emulates the hunting strategy and leadership structure of grey wolves in the wild. It has become more well-known for its ability to resolve a wide range of challenging optimisation issues, such as non-linear inventory issues. Let's examine the GWO algorithm's operation and the justifications for using it to non-linear inventory problems.

### 3.1 Important characteristics of grey wolf optimizer

• Social Structure:

Grey wolves have a social hierarchy in which an alpha pair (leaders), followed by a beta (second in command), a delta (subordinates), and an omega (followers) leads the pack. This hierarchy serves as the model for GWO.

• Mechanism of Hunting:

The search, encirclement, and attack phases of grey wolf hunting are simulated by the algorithm. To address optimisation issues, a mathematical model of this procedure is used.

• Exploration as well as Abuse:

Finding global optima in complicated landscapes requires GWO to properly balance exploration (finding new solutions) and exploitation (improving upon current ones) through its iterative approach.

### 3.2 How come grey wolf optimizer is better for non-linear inventory issues?

• Managing intricacy:

Using conventional optimisation techniques, it can be difficult to model and resolve non-linear inventory problems since they frequently contain complex linkages and constraints. GWO is suited for these issues due to its proficiency in navigating intricate search environments.

• Adaptability:

GWO is adaptable to several non-linear inventory problem types, including multi-objective issues, because it can handle a broad range of objective functions and constraints.

• Global Optimisation:

In non-linear situations, where the solution landscape may have several peaks and valleys, GWO's ability to avoid local optima and look for global solutions is crucial.

• Strong Performance:

The algorithm has proven to be a dependable option for inventory management scenarios due to its consistent performance in a range of optimisation issues.

From the above point of view, we have now employed the Grey Wolf Optimizer (GWO) meta-heuristic approach to solve the six problems mentioned above. It is a well-known, newly created, and widely used optimisation method. Grey Wolf Optimisation (GWO) is a metaheuristic algorithm that draws inspiration from the hunting and social order of grey wolves. An extensive range of optimisation issues, including those involving inventory management, are resolved by this algorithm. The following justifies the possibility that GWO is a suitable option for resolving inventory problems.

(i) GWO is an efficient optimisation algorithm that has been shown to perform well on a variety of optimisation problems, including complex inventory management problems.
(ii) Compared to single-point algorithms, GWO can explore the solution space more completely because it is a population-based approach. This is crucial for inventory management in particular, as there are frequently several ideal solutions that must be found.
(iii) GWO is a dependable technique that performs well on noisy and inconsistent data. This holds particularly true for inventory control, where uncertainty can be introduced into the system by lead times, demand, and supply chain interruptions.
(iv) GWO is easy to utilise and flexible enough to meet the specific needs of the problem. It is simple to include restrictions like safety stock levels, lead times, and inventory capacity into the algorithm.

In summary, we may conclude that, GWO is a powerful optimisation algorithm that can be a great choice for solving inventory related problems. It is very much useful technique to handle uncertainty, integrate restrictions, and explore the solution space rapidly. The details discussion about GWO is provided in solution procedure section.

## 4 Solution procedure

In this section, we have discussed about GWO technique.

### 4.1 inspiration

**Fig. 8:**
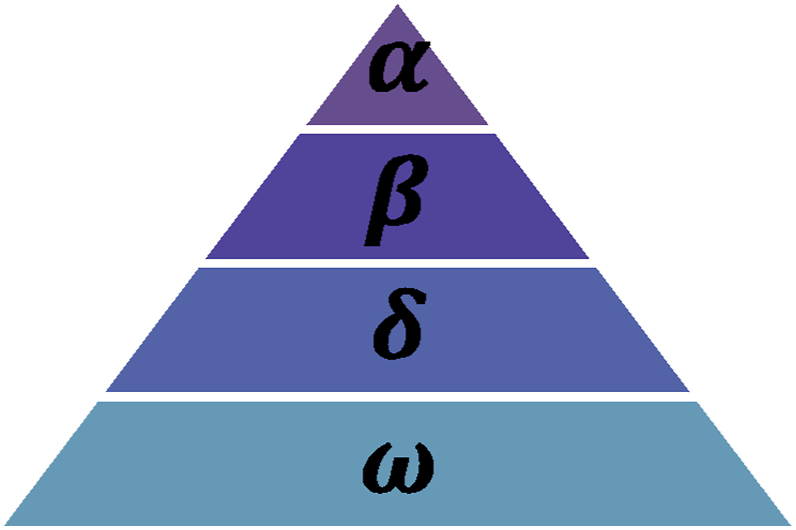
Structure of grey wolf (dominance decreases from top down) (by Mirjalili et al. [91]).

The canid family includes the grey wolf (Canis lupus). Because they are the top predators in the food chain, grey wolves are regarded as apex predators. Usually, grey wolves prefer to live in packs. Each group usually consists of five to twelve individuals. Their very tight social supremacy structure is fascinatingly depicted in Fig. 8.

Alphas or heads, can be either female or male. Alpha is primarily responsible for determining when to wake up, where to sleep, and how to hunt. The pack needs to do as instructed by the alpha. Nonetheless, an alpha can also behave democratically by allowing the group's other wolves to take the lead. When they get together, the entire pack keeps their tails pointing downward in submission to the Alpha. Due to the expectation that the group Mech [92] will obey him, the alpha wolf is often referred to as the dominating wolf. Alpha wolves are the only ones permitted to mate. It is noteworthy to observe that generally the alpha is the pack member who is best at leading the group rather than the one with the highest physical strength. This proves that organisation and self-control are far more important to a pack than size. In the hierarchy of grey wolves, beta is the next rank. The alpha's subordinates, or betas, assist him in decision-making and other pack operations. The beta wolf, who can be either female or male, is most likely the greatest contender to succeed as alpha should one of the other wolves pass away or become too old. In addition to giving orders to the other subordinate wolves, the beta wolf also has a duty of deference to the alpha. It serves as the pack's disciplinarian in addition to being the alpha's counsellor. In addition to providing input to the alpha, the beta serves to reinforce the alpha's instructions across the pack.

The lowest ranking grey wolf is called Omega. The one who does the crime is the omega. Omega wolves must always have space for other dominant wolves. These are the last wolves that are allowed to eat. Although the omega is a relatively insignificant member of the pack, it has been noticed that when the omega disappears, the pack as a whole becomes internally conflicted and has issues. This is the outcome of the furious and violent outbursts of wolves from the omega (s). This keeps the pack peaceful and reserve the social hierarchy of wolf. The pack's babysitters are sometimes the omega.

A wolf that is neither an omega, beta, or alpha is called as a "subordinate" (or delta in other instances). Alpha and beta wolves control delta wolves, are the dominant group among omega wolves. This category includes hunters, caretakers, seniors, scouts, and sentinels. Scouts are responsible for monitoring the area's borders and warning the group of any possible threats. Sentinels protect and maintain the safety of the pack. Elders are older wolves who have held alpha or beta position in the past. Hunters help the betas and alphas hunt and gather food for the group. The sick, injured, and weak wolves in the pack are likewise under the care of their carers.

Hunting of a prey in a group is another noteworthy an element of wolves' social behaviour, according to their hierarchy of social behaviour. According to Muro et al. [93], the main phases of hunting grey wolves are as follows.

**Fig. 9:**
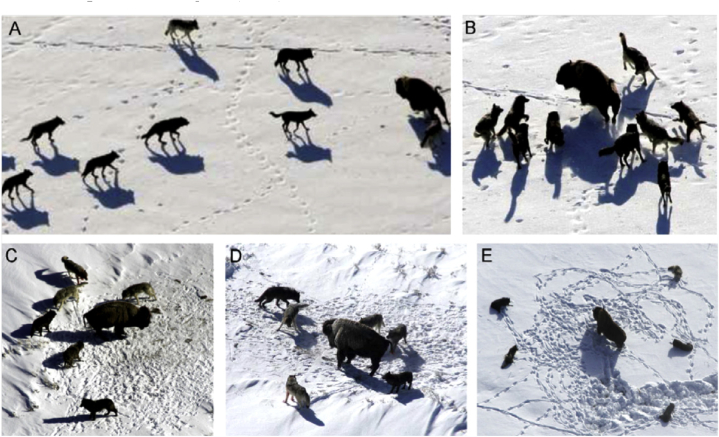
Grey wolves engage in the following hunting behaviours: (A) tracking, pursuing, and approaching prey; (B–D) harassing, encircling, and pursuing; (E) attacking immobile situations (Muro et al. [93], Mirjalili et al. [91]).

➢ Pursuing, tracking, and approaching the target.
➢ Until it stops moving, the target is followed, encircled, and tormented.
➢ Attack the prey directly.
➢ These actions are depicted in Fig. 9 (A-E).

### 4.2 Mathematical representation

This part of the article provides mathematical formulas for locating, encircling, and hunting prey in addition to the social structure. Next, the algorithm known as GWO is described.

#### 4.2.1 Social structure

The structure of Wolf's alpha (a) is simulated analytically and then generated GWO using the best suited solution, or the alpha. As a result, such designations are assigned to the second and third-best responses, beta (b) and delta (d). The final possible response is thought to be omega (x). When it comes to hunting (optimisation), this algorithm follows a guidelines according to alpha (a) first then beta (b) and in the last delta (d). Following these trio of wolves are the omega (x) wolves.

#### 4.2.2 Prey encircling

Grey wolves hunt by circling their prey, as was previously mentioned. The encircling behaviour can be correctly predicted by using the following equations:

(13) $${\vec{E}}_{c}=\left|\vec{L}{\vec{U}}_{p}\left(t\right)-\vec{U}\left(t\right)\right|$$

(14) $$\vec{U}\left(t+1\right)={\vec{U}}_{p}\left(t\right)-\vec{M}.{\vec{E}}_{c}$$

where *t* represent present iteration, $\vec{L}$ and $\vec{M}$ be the coefficient of equations (13), (14). the prey's location vector ${\vec{U}}_{p}\left(t\right)$ and Grey location vector $\vec{U}\left(t\right)$.

The calculation of the vector $\vec{L}$ and $\vec{M}$ are done by using equations (15), (16) in the following way:

(15) $$\vec{M}=2\vec{l}.{\vec{r}}_{1}-\vec{l}$$

(16) $$\vec{L}=2{\vec{r}}_{2}$$

where, during iterations, $\vec{l}$ be reduced 2 to 0 and, ${\vec{r}}_{1}$, ${\vec{r}}_{2}$ be generated randomly in [0,1].

#### 4.2.3 Prey hunting

The capacity to locate prey and encircle them is possessed by grey wolves. In most cases, the alpha leads the search. Hunting may also be periodically practised by the beta and delta. We do not, however, know where the ideal location is in a hypothetical search space (prey). To simulate the hunting behaviour of grey wolves numerically, we suppose that alpha (the best candidate solution), beta, and delta have greater knowledge of potential prey locations. In order to force the other search agents, including the omegas, to update their locations in accordance with the position of the best search agents, we save the first three best solutions we have found thus far. The update of the position are being calculated by using the following formula presented in equations (17), (18), (19):

(17) $${\vec{E}}_{ca}=\left|{\vec{L}}_{a}{\vec{U}}_{a}\left(t\right)-\vec{U}\left(t\right)\right|,{\vec{E}}_{cb}=\left|{\vec{L}}_{b}{\vec{U}}_{b}\left(t\right)-\vec{U}\left(t\right)\right|,{\vec{E}}_{cd}=\left|{\vec{L}}_{d}{\vec{U}}_{d}\left(t\right)-\vec{U}\left(t\right)\right|$$

(18) $${\vec{U}}_{1}\left(t+1\right)={\vec{U}}_{a}\left(t\right)-{\vec{M}}_{a}.{\vec{E}}_{ca},{\vec{U}}_{2}\left(t+1\right)={\vec{U}}_{b}\left(t\right)-{\vec{M}}_{b}.{\vec{E}}_{cb},{\vec{U}}_{3}\left(t+1\right)={\vec{U}}_{d}\left(t\right)-{\vec{M}}_{d}.{\vec{E}}_{cd}$$

(19) $$\vec{U}\left(t+1\right)=\frac{{\vec{U}}_{1}\left(t+1\right)+{\vec{U}}_{2}\left(t+1\right)+{\vec{U}}_{3}\left(t+1\right)}{3}$$

#### 4.2.4 Prey attacking

The grey wolves attack their prey when, as said earlier, it stops and wolfs are break up to hunt. By lowering The significance of $\vec{l}$, anyone could model the pursuit of the prey with mathematical methods. Keep in mind that an also reduces the fluctuation range of $\vec{M}$. Or to put it another way, $\vec{M}$ is a value chosen randomly with the range [−2 $\vec{l}$, 2 $\vec{l}$] where a falls between 2 and 0 over the course of the iterations. Any stance between a prayer and a search agent, it is possible to determine a search agent's present location and the prey's position when $\vec{M}$’s random values are in the range [−1, 1]. If $\left|\vec{M}\right|<1$ then the prey is attacked by the wolves.

#### 4.2.5 Prey searching

Alpha, beta, and delta positions are where grey wolves find prey most frequently. They split off to locate prey; they assemble in together in order to hitting an individual. Persuade the agent research for prey in order to went somewhere else, here it is use $\vec{M}$ in order to mathematically indicate a divergence and random number is generated which is greater than 1 or less than −1. This promotes curiosity and enables a global search for the GWO algorithm. If $\vec{M}$ > 1, grey wolves need to separate from their prey in order to locate more advantageous prey. The component of $\vec{L}$ GWO also promotes research. According to Equation (16), the vector's random values fall between 0 and 2. This section provides randomised prey weights that can be applied to enhance stochastically ($\vec{L}$ > 1) or decrease ($\vec{L}$ < 1) the part of the distance that the prey contributed in Eq. (13).This helps GWO display a more erratic behaviour throughout optimisation, emphasising exploration and avoiding local optima. It is to be mentioned that the values of $\vec{M}$, this decrease in $\vec{L}$ which is nonlinear. We intentionally make $\vec{L}$ always provide the value randomly in order to emphasise exploration in both the first and last rounds of the process. This component is quite helpful when there is stagnation of the premature convergence, particularly in the most recent iterations.

The influence of natural impediments on advancing prey can alternatively be thought of as the $\vec{L}$ vector. In general, wolves face challenges in nature while travelling their hunting trails, which seriously impairs their capacity to approach prey quickly and easily. This is how the vector $\vec{L}$ operates. A wolf can randomly give its give their prey something to weigh which create more harder for predatory wolves to capture, or move in opposite direction, depending on where it is positioned.

In conclusion, the GWO algorithm creates a population of grey wolves at random as the initial phase in the search process, or prospective solutions. The alpha, beta, and delta wolves calculate the potential position of the prey across several iterations. Every potential reaction modifies The distance of the prey from it. To emphasise the significance of research and development, $\vec{l}$ is decreased from the initial value 2 to 0. According to the condition $\left|\vec{M}\right|>1$ or $\left|\vec{M}\right|<1$, wolf move opposite direction or attached to the prey, respectively. This algorithm is terminated when one or more end criteria are met. The pseudo code and details discussions are available in Mirjalili et al. [91].

## 5 Numerical example

To illustrate and solve six problems, six examples are considered for each problem and solved with the help of GWO in MATLAB programming interface and compared the results with some other popular metaheuristic algorithms. The values of the inventory parameters are taken hypothetically. However it is seems to be realistic.

### 5.1 Example-1

Different system parameters values are assumed hypothetical. However, these values are seems to be realistic. For each problem, we have used an example in order to solve the respective optimisation problem. In addition, we have plotted the 3D figures for each objective function for respective examples. The values of the different parameters are given below:

$$\begin{array}{c} \lambda =150;\sigma =0.9;\tau =2;\upsilon =0.5;\;c_{h}=2.8;\;K=300;\;c_{1}=10;c_{2}=0.5;\gamma =0.2;\varphi_{e}=0.04;\varphi_{c}=0.09\text{;}\\ S_{1}=125;\;\xi =0.6;\mu =0.85\text{;} \end{array}$$

Table 1 below displays the ideal solutions for Example 1.

**Table 1:** Best outcomes discovered for Example-1.

Variables/unknown parameters	Optimal values obtain from (GWO)	Optimal values obtain from (WOA)	Optimal values obtain from (AEFA)
$s_{p}$	$ 90.6782	$ 90.4532	$ 90.5732
T	1.4228 years	1.4438 years	1.4448 years
S	99.8862 unit	99.7861 unit	99.7552 unit
$ATP_{1.1}\left(s_{p},T\right)$	5298.0314	5298.0104	5298.0254

**Fig. 10:**
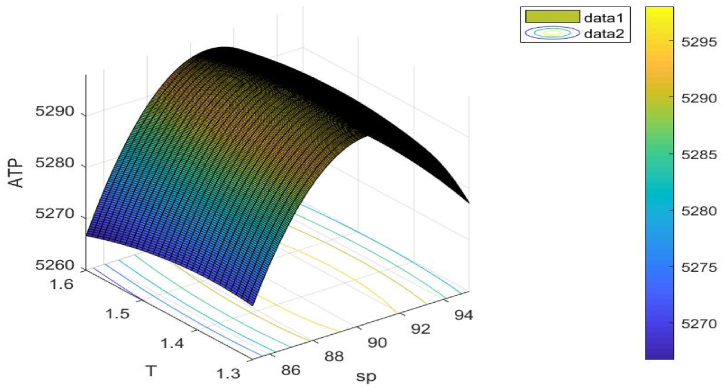
Concavity of problem 1's objective function with regard to Example 1.

Fig. 10 illustrates the objective function's concavity. The choice variable $s_{p}$**,** T is taken into consideration when drawing this figure.

### 5.2 Example-2

There are assumptions underlying the values of the different system parameters. Still, these seem like reasonable figures. For each problem, we have employed an example to assist us in solving the related optimisation problem. We also drew the 3D figures for the appropriate examples of each objective function. The values for the different parameters are listed below: $\begin{array}{l} \lambda =150;\sigma =0.9;\tau =2;\upsilon =0.5;\;c_{h}=2.8;\;K=300;\;c_{1}=10;c_{2}=0.5;\gamma =0.2;\varphi_{e}=0.04;\varphi_{c}=0.09\text{;}\\ S_{1}=125;\;\xi =0.7;\mu =0.85\text{;} \end{array}$.

Following Table 2, the ideal outcomes of Example 2 are displayed.

**Table 2:** Best outcomes discovered for Example 2.

Variables/unknown parameters	Optimal values obtain from (GWO)	Optimal values obtain from (WOA)	Optimal values obtain from (AEFA)
$s_{p}$	$ 90.6782	$ 90.4532	$ 90.5732
T	1.4228 years	1.4438 years	1.4448 years
S	99.8862 unit	99.7865 unit	99.7553 unit
$ATP_{1.1}\left(s_{p},T\right)$	5298.0314	5298.0104	5298.0254

**Fig. 11:**
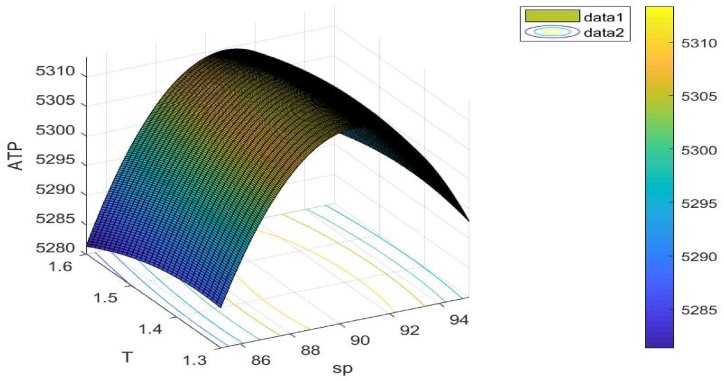
Concavity of problem 2's objective function with regard to Example 2.

Fig. 11 illustrates the objective function's concavity. The choice variable $s_{p}$**,** T is taken into consideration when drawing this figure.

### 5.3 Example-3

There are assumptions underlying the values of the different system parameters. Still, these seem like reasonable figures. For each problem, we have employed an example to assist us in solving the related optimisation problem. We also drew the 3D figures for the appropriate examples of each objective function. The values for the different parameters are listed below: $\begin{array}{l} \lambda =150;\sigma =0.9;\tau =2;\upsilon =0.5;\;c_{h}=2.8;\;K=300;\;c_{1}=10;c_{2}=0.5;\gamma =0.2;\varphi_{e}=0.04;\varphi_{c}=0.09\text{;}\\ S_{1}=125;\;\xi =0.8;\mu =0.9;\nu =0.2\text{;} \end{array}$.

Following Table 3, the ideal outcomes of Example 3 are displayed.

**Table 3:** Best outcomes discovered for Example 3.

Variables/unknown parameters	Optimal values obtain from (GWO)	Optimal values obtain from (WOA)	Optimal values obtain from (AEFA)
$s_{p}$	$ 90.9126	$ 90.4532	$ 90.5732
T	1.4152 years	1.4438 years	1.4448 years
S	99.1758unit	99.7861 unit	99.7552 unit
$ATP_{1.1}\left(s_{p},T\right)$	5216.0926	5216.0914	5216.0917

**Fig. 12:**
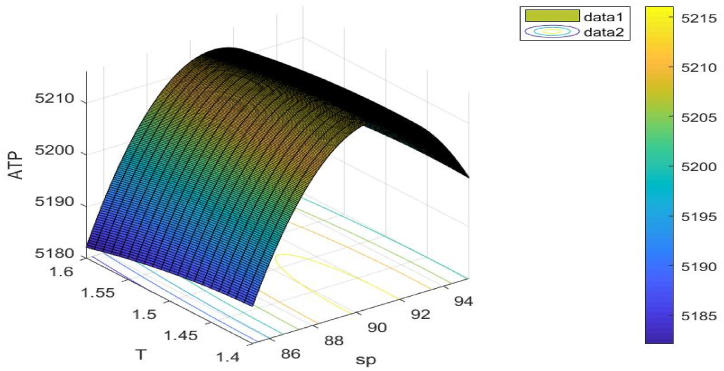
Concavity of problem 3's objective function with regard to Example 3.

Fig. 12 illustrates the objective function's concavity. The choice variable $s_{p}$**,** T is taken into consideration when drawing this figure.

### 5.4 Example-4

There are assumptions underlying the values of the different system parameters. Still, these seem like reasonable figures. For each problem, we have employed an example to assist us in solving the related optimisation problem. We also drew the 3D figures for the appropriate examples of each objective function. The values for the different parameters are listed below: $\begin{array}{l} \lambda =150;\sigma =0.9;\tau =2;\nu =0.5;\;c_{h}=2.8;\;K=300;\;c_{1}=10;c_{2}=0.5;\gamma =0.2;\varphi_{e}=0.04;\varphi_{c}=0.09\text{;}\\ S_{1}=125;\;\xi =0.4;\mu =0.9\text{;} \end{array}$.

Following Table 4, the ideal outcomes of Example 4 are displayed.

**Table 4:** Best outcomes discovered for Example 4.

Variables/unknown parameters	Optimal values obtain from (GWO)	Optimal values obtain from (WOA)	Optimal values obtain from (AEFA)
$s_{p}$	$ 90.3022	$ 90.4532	$ 90.5732
T	2.6057years	2.6177years	2.6075 years
S	184.0344 unit	184.0432 unit	184.0442 unit
$ATP_{1.1}\left(s_{p},T\right)$	5310.8880	5310.8670	5310.8775

**Fig. 13:**
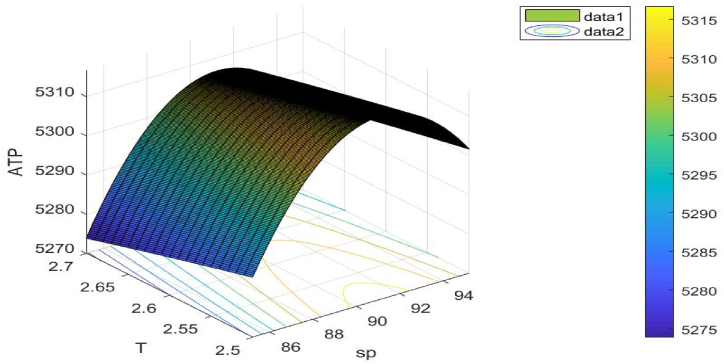
Concavity of problem 4's objective function with regard to Example 4.

Fig. 13 illustrates the objective function's concavity. The choice variable $s_{p}$**,** T is taken into consideration when drawing this figure.

### 5.5 Example-5

There are assumptions underlying the values of the different system parameters. Still, these seem like reasonable figures. For each problem, we have employed an example to assist us in solving the related optimisation problem. We also drew the 3D figures for the appropriate examples of each objective function. The values for the different parameters are listed below: $\begin{array}{l} \lambda =150;\sigma =0.9;\tau =2;\upsilon =0.5;\;c_{h}=2.8;\;K=300;\;c_{1}=10;c_{2}=0.5;\gamma =0.2;\varphi_{e}=0.04;\varphi_{c}=0.09\text{;}\\ S_{1}=125;\;\xi =0.3;\mu =0.9;\nu =0.2 \end{array}$.

Following Table 5, the ideal outcomes of Example 5 are displayed.

**Table 5:** Best outcomes discovered for Example 5.

Variables/unknown parameters	Optimal values obtain from (GWO)	Optimal values obtain from (WOA)	Optimal values obtain from (AEFA)
$s_{p}$	$ 90.8082	$ 90.7532	$ 90.7732
T	1.7947years	1.8138 years	1.8148 years
S	125.9366unit	125.7345 unit	125.8845 unit
$ATP_{1.1}\left(s_{p},T\right)$	5307.2733	5307.2622	5307.2623

**Fig. 14:**
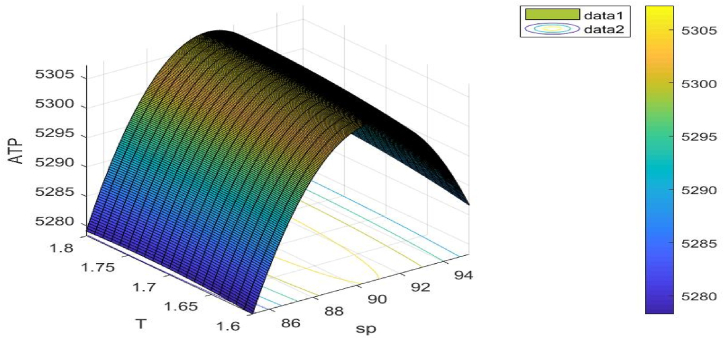
Concavity of problem 5's objective function with regard to Example 5.

Fig. 14 illustrates the objective function's concavity. The choice variable $s_{p}$**,** T is taken into consideration when drawing this figure.

### 5.6 Example-6

The values used for the different inventory parameters are artificial. Yet it seems like these values are reasonable. For the purpose of resolving the corresponding optimisation issue, we have included an example for each problem. For each objective function for the associated samples, we have also drawn 3D figures. This is a list of all the parameters' values:

$$\begin{array}{c} \lambda =150;\sigma =0.9;\tau =2;\upsilon =0.5;\;c_{h}=2.8;\;K=300;\;c_{1}=10;c_{2}=0.5;\gamma =0.2;\varphi_{e}=0.04;\varphi_{c}=0.09\text{;}\\ S_{1}=125;\;\xi =0.2;\mu =0.9;\nu =0.2\text{.} \end{array}$$

Following Table 6, the ideal outcomes of Example 6 are displayed.

**Table 6:** Best outcomes discovered for Example 6.

Variables/unknown parameters	Optimal values obtain from (GWO)	Optimal values obtain from (WOA)	Optimal values obtain from (AEFA)
$s_{p}$	$ 90.9253	$ 90.4532	$ 90.5732
T	1.4131 years	1.4432 years	1.4441 years
S	99.0077 unit	99.0076 unit	99.0075 unit
$ATP_{1.1}\left(s_{p},T\right)$	5221.5811	5221.5800	5221.5801

**Fig. 15:**
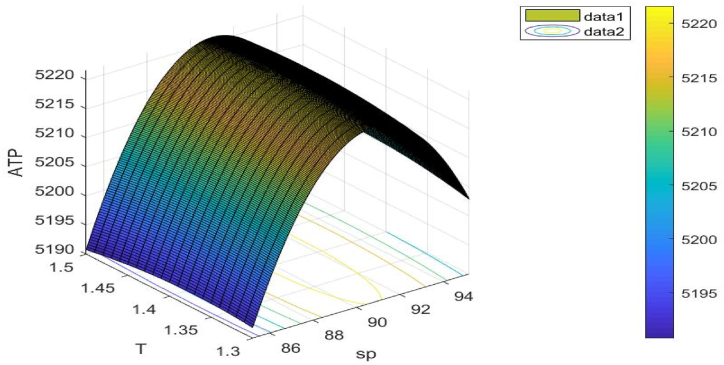
Concavity of problem 6's objective function with regard to Example 6.

Fig. 15 illustrates the objective function's concavity. The choice variable $s_{p}$**,** T is taken into consideration when drawing this figure.

## 6 Sensitivity analysis

To determine how various inventory factors affect average profit (ATP), selling price (p) and business period (T), post optimality analysis are executed and presented in Table 7.

**Table 7:** Post optimality analysis of Example 1.

Inventory parameters	Decision variables/average profit	Change in percentages				
		−20 %	−10 %	0 %	20 %	10 %
λ	ATP	−25.5767	−15.95378	0	14.5628	26.3274
	S	−14.5324	−9.5432	0	10.5241	15.5432
	T	−14.6911	−8.57091	0	8.42076	16.9651
	$s_{p}$	−21.2913	−11.9112	0	10.5414	19.7615
σ	ATP	5.3468	3.6437	0	−4.6125	−5.4218
	S	−10.5214	−7.3452	0	6.5241	11.5332
	T	3.6943	1.5702	0	−2.3241	−4.5231
	$s_{p}$	−12.4537	−6.9112	0	8.7424	14.5617
τ	ATP	−1.5767	−0.6437	0	0.5742	1.3274
	S	2.5324	1.5432	0	−1.5241	2.7432
	T	1.6911	0.5709	0	−0.42076	−1.9651
	$s_{p}$	−1.2913	−0.9112	0	1.5414	2.7615
υ	ATP	5.3468	3.6437	0	−4.6125	−5.4218
	S	−10.5214	−7.3452	0	6.5241	11.5332
	T	3.6943	1.5702	0	−2.3241	−4.5231
	$s_{p}$	−12.4537	−6.9112	0	8.7424	14.5617
$c_{1}$	ATP	−1.6765	−0.3437	0	0.4742	1.5274
	S	2.6324	1.7432	0	−1.8241	2.9432
	T	1.4911	0.6709	0	−0.52076	−1.8651
	$s_{p}$	−1.3913	−0.7112	0	1.6414	2.8615
$c_{2}$	ATP	−1.5767	−0.6437	0	0.5742	1.3274
	S	2.5324	1.5432	0	−1.5241	2.7432
	T	1.6911	0.5709	0	−0.42076	−1.9651
	$s_{p}$	−1.2913	−0.9112	0	1.5414	2.7615
$c_{h}$	ATP	−1.3767	−0.2437	0	0.1742	1.4274
	S	2.6324	1.6432	0	−1.3241	2.3432
	T	1.6911	0.5709	0	−0.42076	−1.9651
	$s_{p}$	−1.4913	−0.8112	0	1.6414	2.8615
μ	ATP	−25.7767	−15.65378	0	14.4628	26.2274
	S	−14.4324	−9.3432	0	10.2241	15.1432
	T	−14.5911	−8.67091	0	8.72076	16.8651
	$s_{p}$	−21.2913	−11.9112	0	10.5414	19.7615
ξ	ATP	4.3468	2.6437	0	−3.6125	−4.4218
	S	−11.5214	−8.3452	0	7.5241	10.5332
	T	3.7943	1.6702	0	−2.4241	−4.6231
	$s_{p}$	−11.4537	−5.9112	0	7.7424	13.5617
ν	ATP	5.3468	3.6437	0	−4.6125	−5.4218
	S	−11.5214	−6.3452	0	5.5241	10.5332
	T	3.6943	1.5702	0	−2.3241	−4.5231
	$s_{p}$	−11.4537	−4.9112	0	7.7424	13.5617

We note the following implications.

(i) From Tables 7 and it is observe that profit per unit (ATP) is highly responsive whereas length of replenishment cycle (T), stock of items (S) and the selling price $\left(s_{p}\right)$ are moderately sensitive against the changes of interval valued initial fixed demand λ,μ.
(ii) From Tables 7 and it can be seen that profit per unit (ATP) is equally sensitive and the selling price $\left(s_{p}\right)$ is equally sensitive but all are reversely effected with the changes of demand parameter σ,ξ, whereas stock of items (S) and the length of replenishment cycle (T) is moderately sensitive.
(iii) From Tables 7 and it is observe that the profit per unit (ATP), length of replenishment cycle (T), stock of items (S) and the selling price $\left(s_{p}\right)$ are less sensitive with respect to rest of the parameters.

### 6.1 Managerial insight

Understanding the expenses related to creating or purchasing the good or service is crucial before deciding on a selling price. This covers both direct costs like labour and supplies as well as indirect costs like utilities, rent, and advertising. A product or service's selling price ought to be determined by what the consumer base is willing to pay. This entails being aware of the value that consumers place on the good or service as well as the costs that rival businesses charge.

A selling price can be determined using a variety of pricing techniques, including value-based pricing, cost-plus pricing, and dynamic pricing. Every strategy has benefits and drawbacks of its own, so the decision regarding which to use should be based on the market, the company's objectives, and the product or service.

The selling price ought to be flexible. It's critical to keep an eye on market conditions and modify prices as needed. Retaining competitiveness and profitability may benefit from this. Lastly, it's critical to convey to clients the value that the good or service offers. This can strengthen customer loyalty and provide justification for a higher selling price. Green products are becoming more and more popular, but before investing in them, it's critical to assess the market. Conduct market research to identify if customers are willing to pay extra for sustainable products and what features they consider to be most important.

### 6.2 Concluding remarks

One appealing approach to inventory management is credit financing. We have created an inventory model in this study that takes price and green level into account when determining demand. The degree of greenness of the product also affects production costs. There is detailed coverage of several scenarios and sub scenarios. Because nonlinearity nature of the goal function, it is challenging task to find the closed form solution of the goal function. Due to this reason, three different meta heuristic algorithms are used to solve the said problem. It is observed that Grey Wolf Optimizar (GWO) performed better than other algorithms. It can be seen from the computational results that GWO outperforms other algorithms. With MATLAB 2018a, the objective function's concavity is visually displayed. This example offers instructions for creating business plans for companies that, in addition to the previously listed industries, manufacture bakeries, pharmacies, cosmetics, cement, chemicals, food products (such as sugar, powder milk), alcoholic beverages, and other goods. The main limitation of this model is to unable to solve the problem analytically.

Anybody can add nonlinear stock dependent demand, use preservation technology, employ carbon cap and trade policy, trade credit (both single and two level), etc. for further exploration. Also, anyone can include interval objective in order to use the soft computing technique to resolve this kind of challenging issue.

## Data availability statement

Data will be made available on request.

## CRediT authorship contribution statement

**Adel Fahad Alrasheedi:** Writing – review & editing, Writing – original draft, Methodology, Investigation, Funding acquisition, Formal analysis, Data curation, Conceptualization.

## Declaration of competing interest

The authors declare the following financial interests/personal relationships which may be considered as potential competing interests:All the authors are declaring the manuscript whose entitled **“Different type of pay later facility for green product with selling price dependent demand using Grey Wolf Optimizer”** for possible publication in your esteemed journal has no conflict interest.
